# Supplementary material for: Digenic inheritance of mutations in EPHA2 and SLC26A4 in Pendred syndrome
Source: Nat Commun. 2020 Mar 12;11:1343. doi: 10.1038/s41467-020-15198-9 (PMC7067772; doi:10.1038/s41467-020-15198-9)
Supplement: Supplementary file 1 — Supplementary Information [file 41467_2020_15198_MOESM1_ESM.pdf]

Supplementary Information

Digenic inheritance of mutations in *EPHA2* and *SLC26A4* in Pendred syndrome

Mengnan Li, Shin-ya Nishio, Chie Naruse, Meghan Riddell, Sabrina Sapski, Tatsuya Katsuno, Takao Hikita, Fatemeh Mizapourshafiyi, Fiona M Smith, Leanne T Cooper, Min Goo Lee, Masahide Asano, Thomas Boettger, Marcus Krueger, Astrid Wietelmann, Johannes Graumann, Bryan W Day, Andrew W Boyd, Stefan Offermanns, Shin-ichiro Kitajiri, Shin-ichi Usami, and Masanori Nakayama

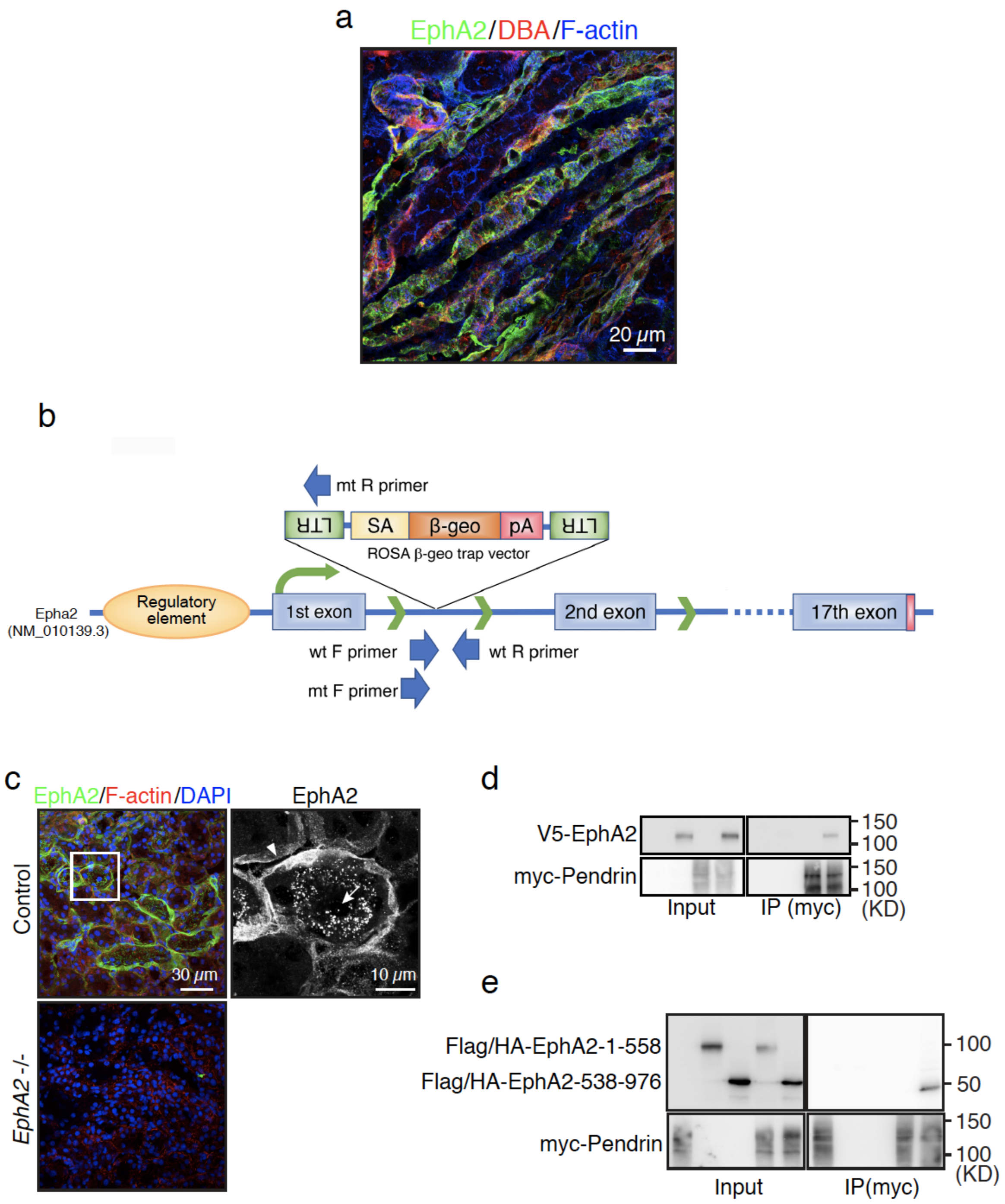

Supplementary Figure 1. Pendrin is a novel binding partner of EphA2. a) EphA2 was expressed in the DBA-positive collecting duct. EphA2, green; DBA, red; Phalloidin (actin), blue. b) Gene targeting strategy of EphA2 KO mice. c) Immunostaining of EphA2 in kidney sections with phalloidin (actin) and DAPI (nucleus). Enlarged images of indicated region in left top panel was shown in the right panel. The arrowhead indicates EphA2 expressed at the basal membrane of the epithelial cells in the collecting duct. The arrow indicates EphA2 showing a dot-like pattern around the apical surface of the epithelial cells. d) Immunoprecipitation of V5-tagged EphA2 with myc-pendrin overexpressed in HEK293T cells. e) Immunoprecipitation of truncated forms of EphA2 with myc-pendrin. Indicated truncated forms of EphA2 were overexpressed in HEK293T cells.

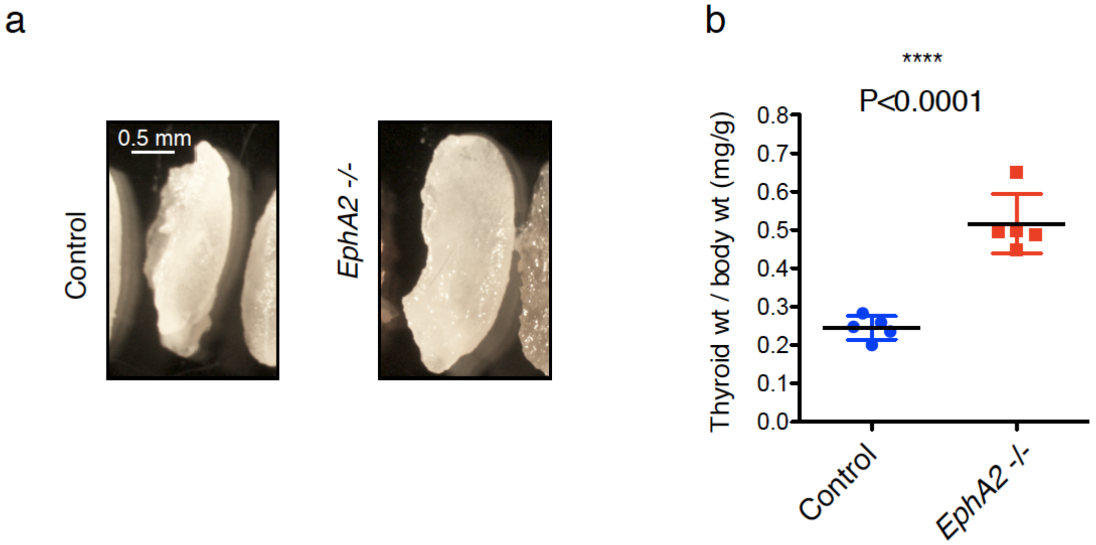

Supplementary Figure 2. Thyroid goiter of EphA2 KO mice. a) View of the thyroid of control (left) and *EphA2* KO mice (right). b) Relative weight of thyroid normalized to body weight. Lines indicate mean value from 4 animals with SD. P<0.0001 by student t-test. Source data are provided as a Source Data file.

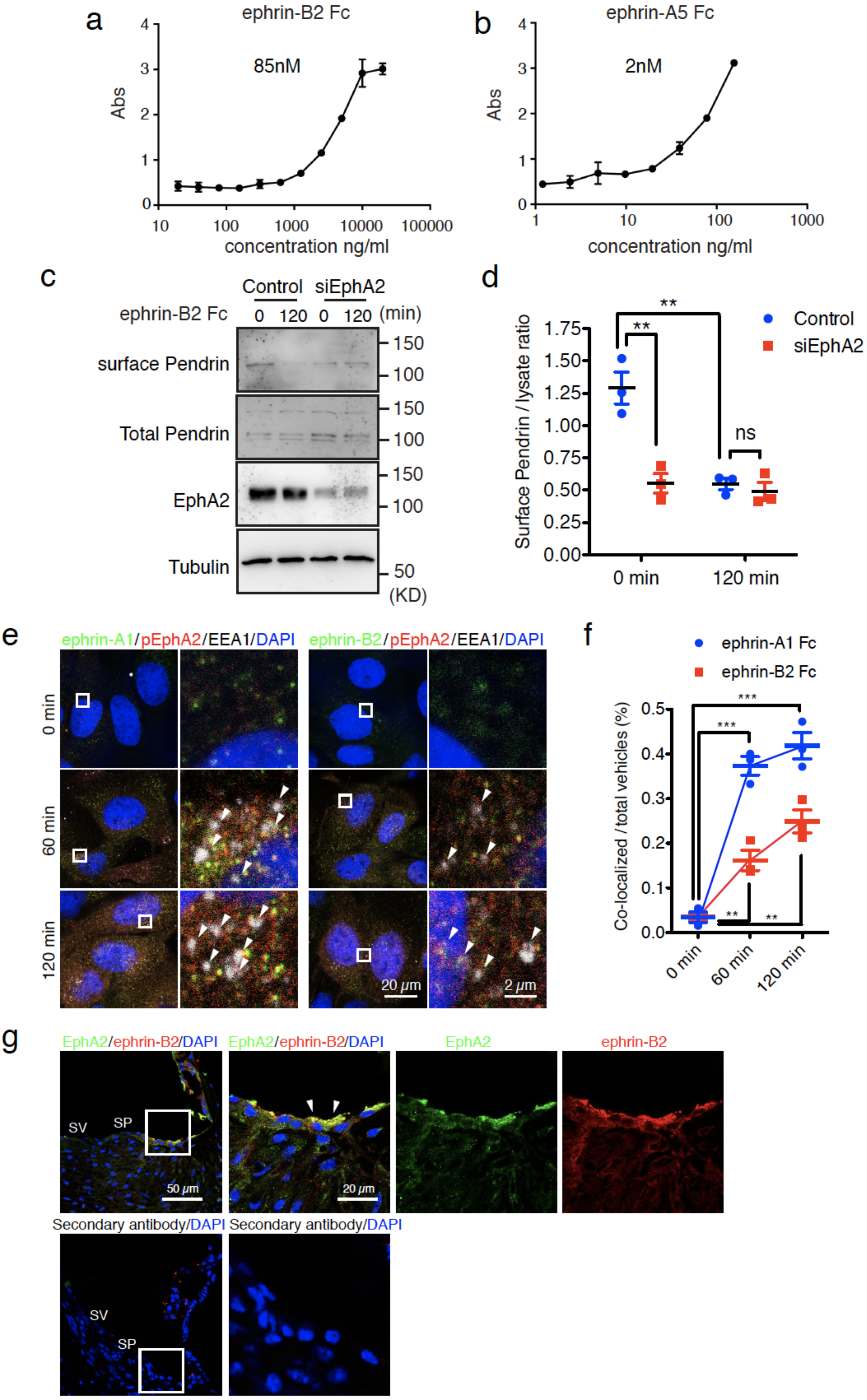
a
ephrin-B2 Fc
85nM
Abs
concentration ng/ml
b
ephrin-A5 Fc
2nM
Abs
concentration ng/ml
c
Control siEphA2
ephrin-B2 Fc 0 120 0 120 (min)
surface Pendrin
Total Pendrin
EphA2
Tubulin
(KD)
d
Control
siEphA2
Surface Pendrin / lysate ratio
0 min
120 min
ns
e
ephrin-A1/pEphA2/EEA1/DAPI
ephrin-B2/pEphA2/EEA1/DAPI
0 min
60 min
120 min
20 μm
2 μm
f
ephrin-A1 Fc
ephrin-B2 Fc
Co-localized / total vehicles (%)
0 min
60 min
120 min
g
EphA2/ephrin-B2/DAPI
EphA2
ephrin-B2
SV
SP
50 μm
20 μm
Secondary antibody/DAPI

Supplementary Figure 3. ephrin-B2 is a functional ligand of EphA2. a, b) KD determination of ephrin-B2 Fc fusion protein (a) or ephrin-A5 Fc fusion protein (b) with EphA2 Fc fusion protein. Calculated affinity binding KD values are shown. c) siRNA against EphA2 was transfected into MDCK II cells. The amount of surface pendrin and total pendrin as well as those of EphA2 was examined. Cells are stimulated with ephrin-B2 Fc fusion protein. d) Quantification of surface pendrin in (c) is shown. Lines indicate mean value from 3 independent experiments with SEM. ** $P<0.01$ by student t-test. e) Co-localization of phospho-EphA2 with EEA1 after ephrin-B2 stimulation. The cells are treated with ephrin-A1 or ephrin-B2 Fc fusion proteins for indicated time. Immunostaining of cells using anti-phospho-EphA2 specific antibody (red) with anti-ephrin-B2 or anti-ephrin-A1 antibody (green), anti-EEA1 antibody (grey) and DAPI (blue, nucleus). f) Quantification of colocalization of phospho-EphA2 with EEA1 in (e) is shown. Lines indicate mean value from 3 independent experiments with SEM. * $P<0.05$, ** $P<0.01$ by student t-test. g) Immunostaining of ephrin-B2 with EphA2 in the inner ear of mice. Green, EphA2; red, ephrin-B2; blue, DAPI. The section stained without primary antibodies was used as a negative control. Source data are provided as a Source Data file.

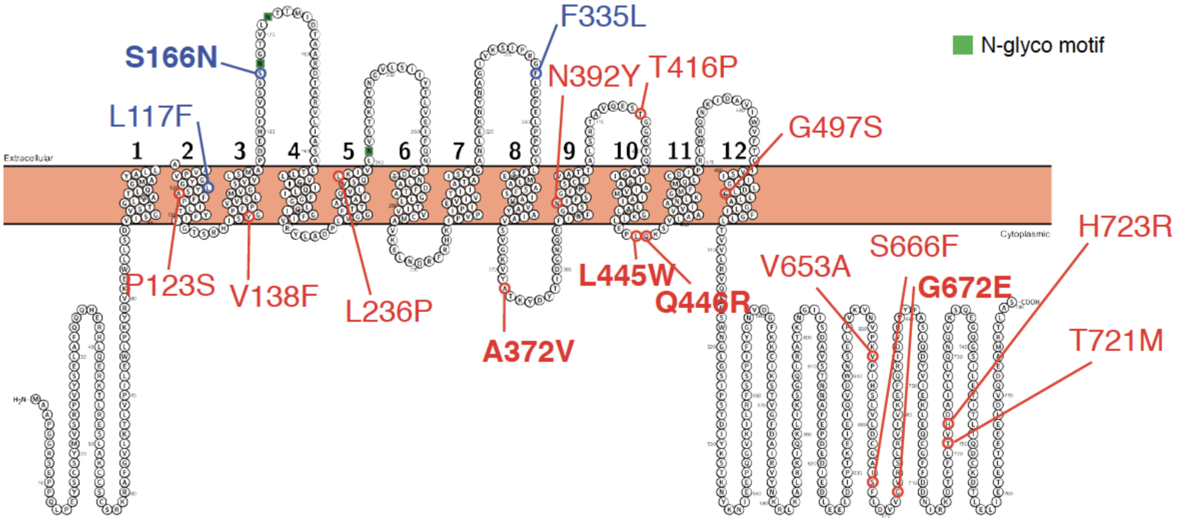

Supplementary Figure 4. Schematic representation of the pendrin mutations identified previously. Red characters indicate known mutations causing mis-localization of pendrin from the plasma membrane, while blue characters indicate mutations showing plasma membrane localization in cultured cells.

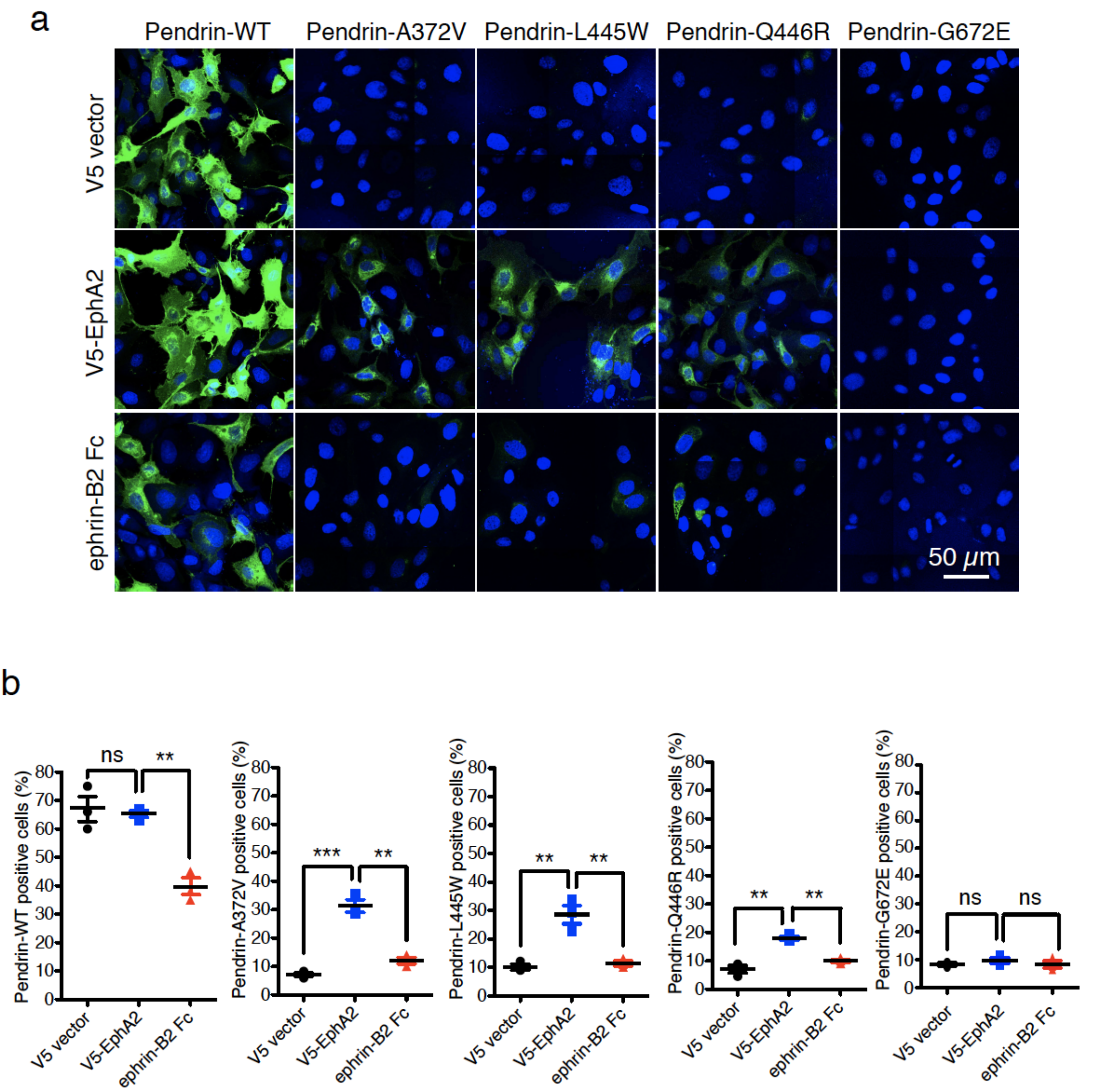

Supplementary Figure 5. Rescue experiment of mutated pendrin with EphA2 overexpression. a) Membrane localized pendrin is shown. Transfected cells were stained with the anti-V5 antibody without permeabilization. After ephrin-B2 Fc-fusion protein treatment, cell surface pendrin was internalized. Green, pendrin; blue, DAPI. b) Membrane localized pendrin positive cells are quantified. Lines indicate mean value from 3 different experiments with SEM. one-way ANOVA; $**p < 0.01$; $(n = 3)$. Source data are provided as a Source Data file.

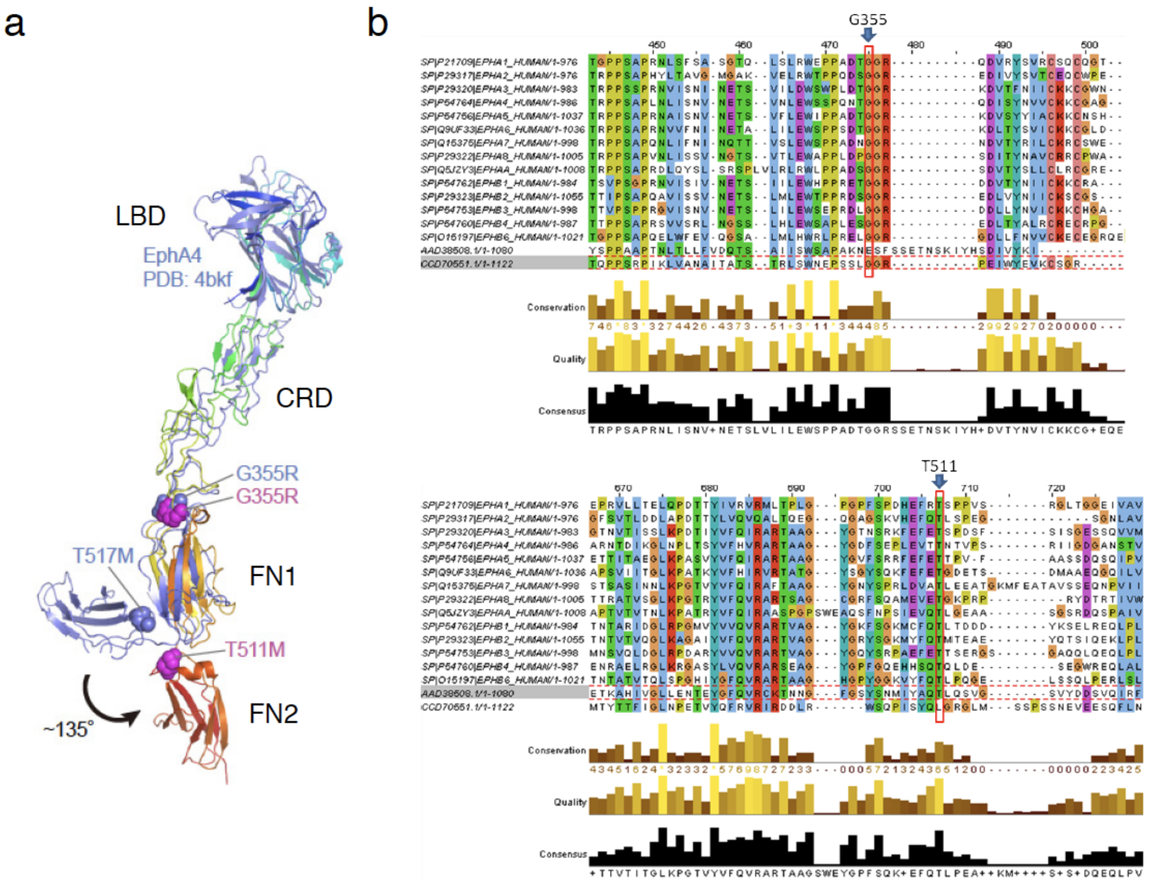

Supplementary Figure 6. EphA2 extracellular domain structure a) Structure of the EphA2 extracellular domain with identified EphA2 mutations from patients. LBD, ligand binding domain; CRD, cysteine-rich domain; FN1, fibronectin1; FN2 fibronectin2. Due to the flexibility in the EphA2 FN1-Fn2 linker region, EphA2 and EphA4 structures were overlaid. b) The sequences of human, C-elegans, and Drosophila Eph RTKs around the identified mutation sites.

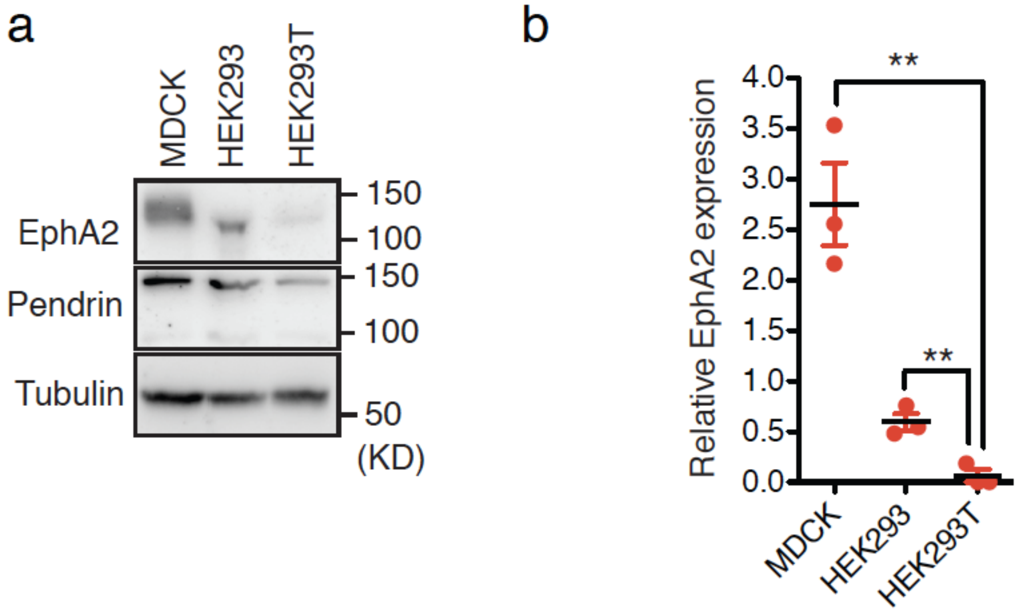

Supplementary Figure 7. Expression of EphA2 in different cell lines a) Western blotting analysis of indicated cultured cell lysate. B) Relative expression of EphA2 in each cell line are shown. Lines indicate mean value from 3 different experiments with SEM. one-way ANOVA; $**p < 0.01$; ($n = 3$). Source data are provided as a Source Data file.

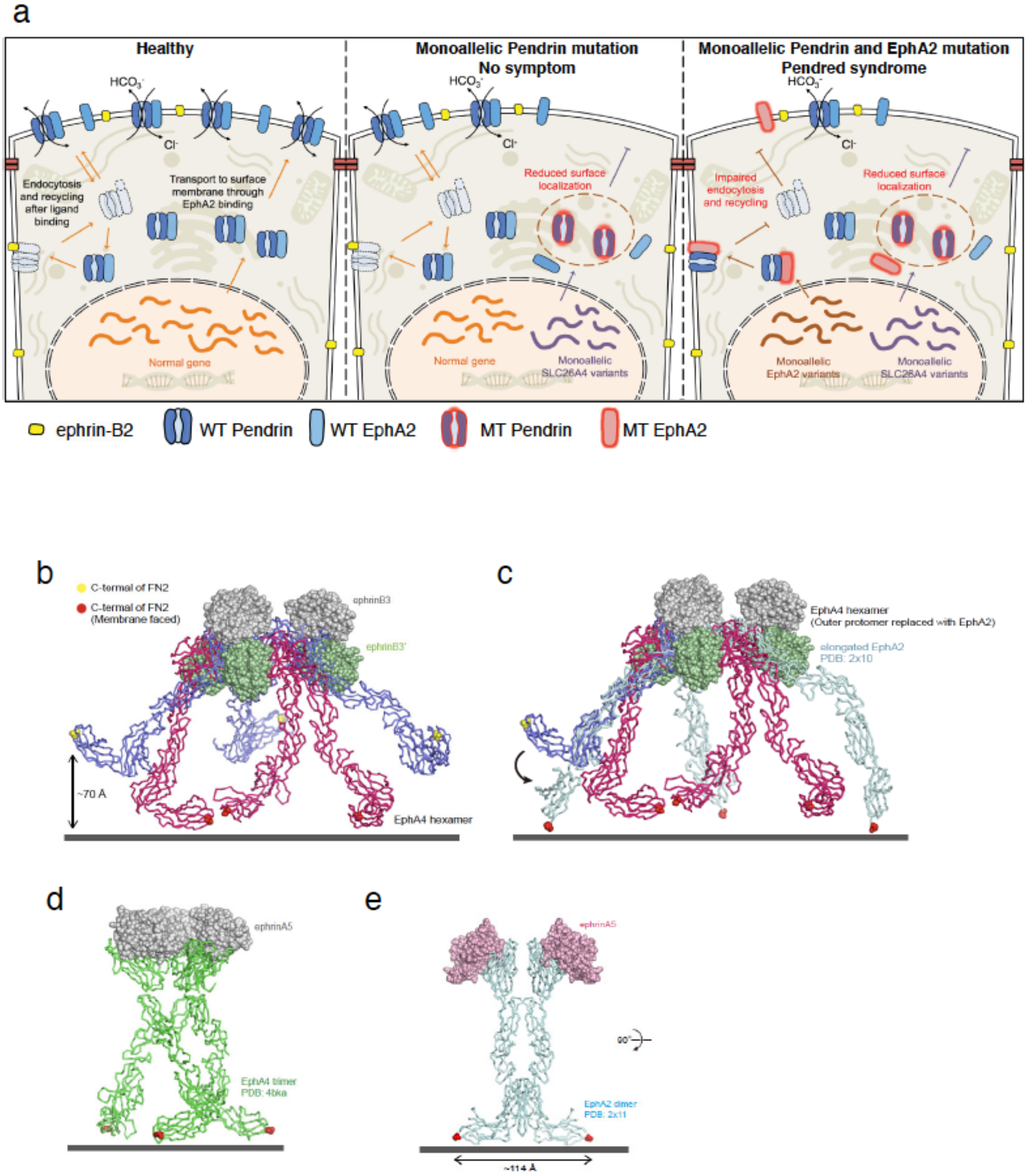

Supplementary Figure 8. EphA2 controls pendrin recruitment to the apical membrane of epithelial cells. a) At cell-to-cell contact sites, ephrin-B2 may induce pendrin internalization via EphA2 to remove the protein complex. Mono-allelic mutation on the *SLC26A4* gene does not cause the symptom, indicating the single allele of the *SLC26A4* gene is sufficient for pendrin function. The pendred syndrome patients carrying the mono-allelic mutation on the SCL26A4 genes with the mono-allelic *EPHA2* mutation may cause mislocalization of pendrin at the cell-to-cell contact sites, resulting in reduced functional pendrin on the apical surface of the epithelial cells. b) Previously shown crystal lattice assemblies compatible with clustering of EphA4 receptors with ephrin-B3. c) Outer protomer of EphA4 was replaced with elongated EphA2. d) Previously shown structure of the EphA4/ephrin-A5 complex. e) Previously shown structure of the EphA2/ephrin-A5 complex.

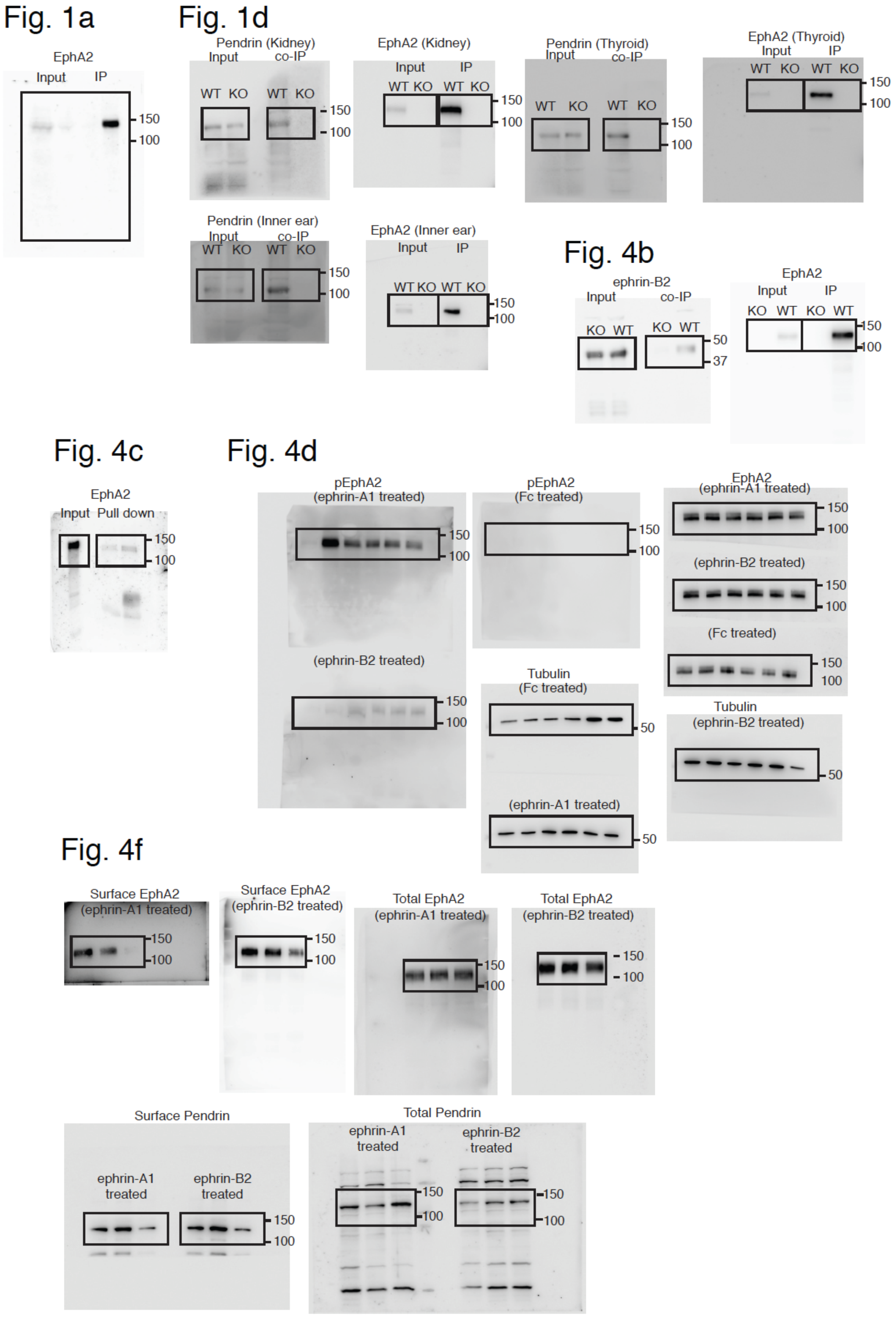

Fig. 1a
EphA2
Input IP
150
100
Fig. 1d
Pendrin (Kidney)
Input co-IP
WT KO WT KO
150
100
EphA2 (Kidney)
Input IP
WT KO WT KO
150
100
Pendrin (Thyroid)
Input co-IP
WT KO WT KO
150
100
EphA2 (Thyroid)
Input IP
WT KO WT KO
150
100
Pendrin (Inner ear)
Input co-IP
WT KO WT KO
150
100
EphA2 (Inner ear)
Input IP
WT KO WT KO
150
100
Fig. 4b
ephrin-B2
Input co-IP
KO WT KO WT
50
37
EphA2
Input IP
KO WT KO WT
150
100
Fig. 4c
EphA2
Input Pull down
150
100
Fig. 4d
pEphA2
(ephrin-A1 treated)
150
100
(ephrin-B2 treated)
150
100
pEphA2
(Fc treated)
150
100
Tubulin
(Fc treated)
50
(ephrin-A1 treated)
50
EphA2
(ephrin-A1 treated)
150
100
(ephrin-B2 treated)
150
100
(Fc treated)
150
100
Tubulin
(ephrin-B2 treated)
50
Fig. 4f
Surface EphA2
(ephrin-A1 treated)
150
100
Surface EphA2
(ephrin-B2 treated)
150
100
Total EphA2
(ephrin-A1 treated)
150
100
Total EphA2
(ephrin-B2 treated)
150
100
Surface Pendrin
ephrin-A1 treated
ephrin-B2 treated
150
100
Total Pendrin
ephrin-A1 treated
ephrin-B2 treated
150
100
150
100

Fig. 5a

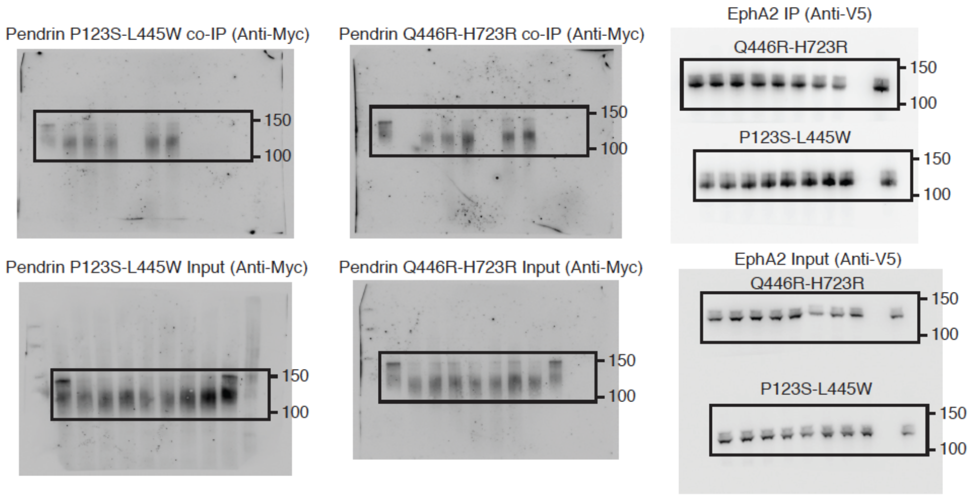

Fig. 5c

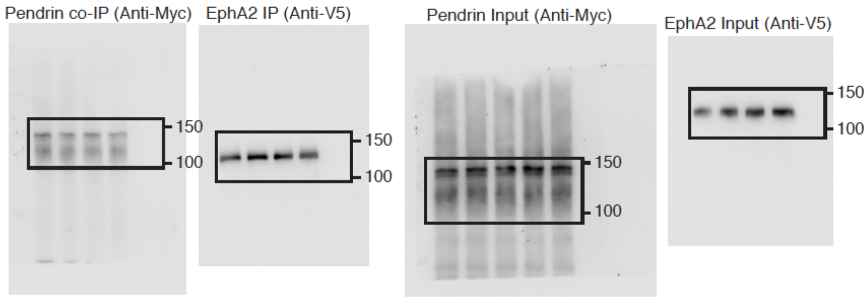

Fig. 5e

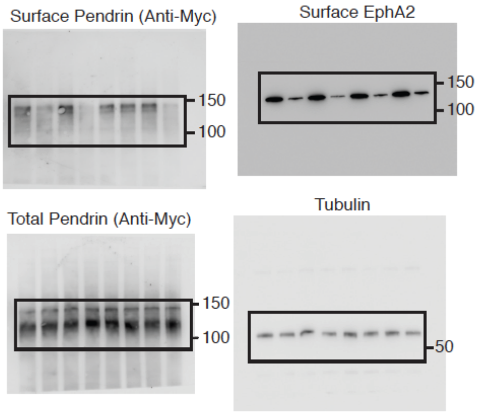

Fig. 7a

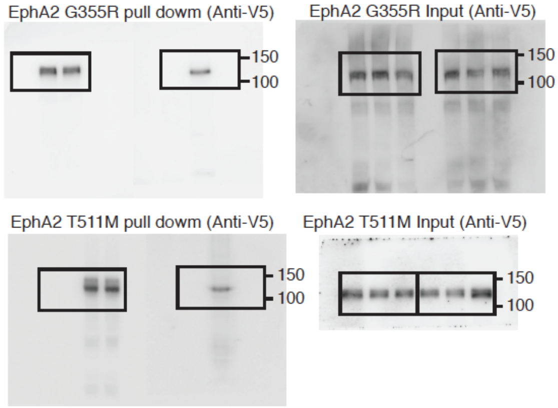

Fig. 7b

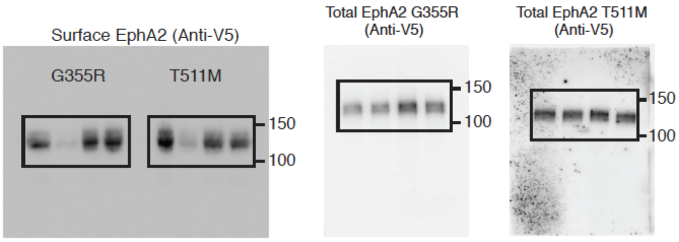

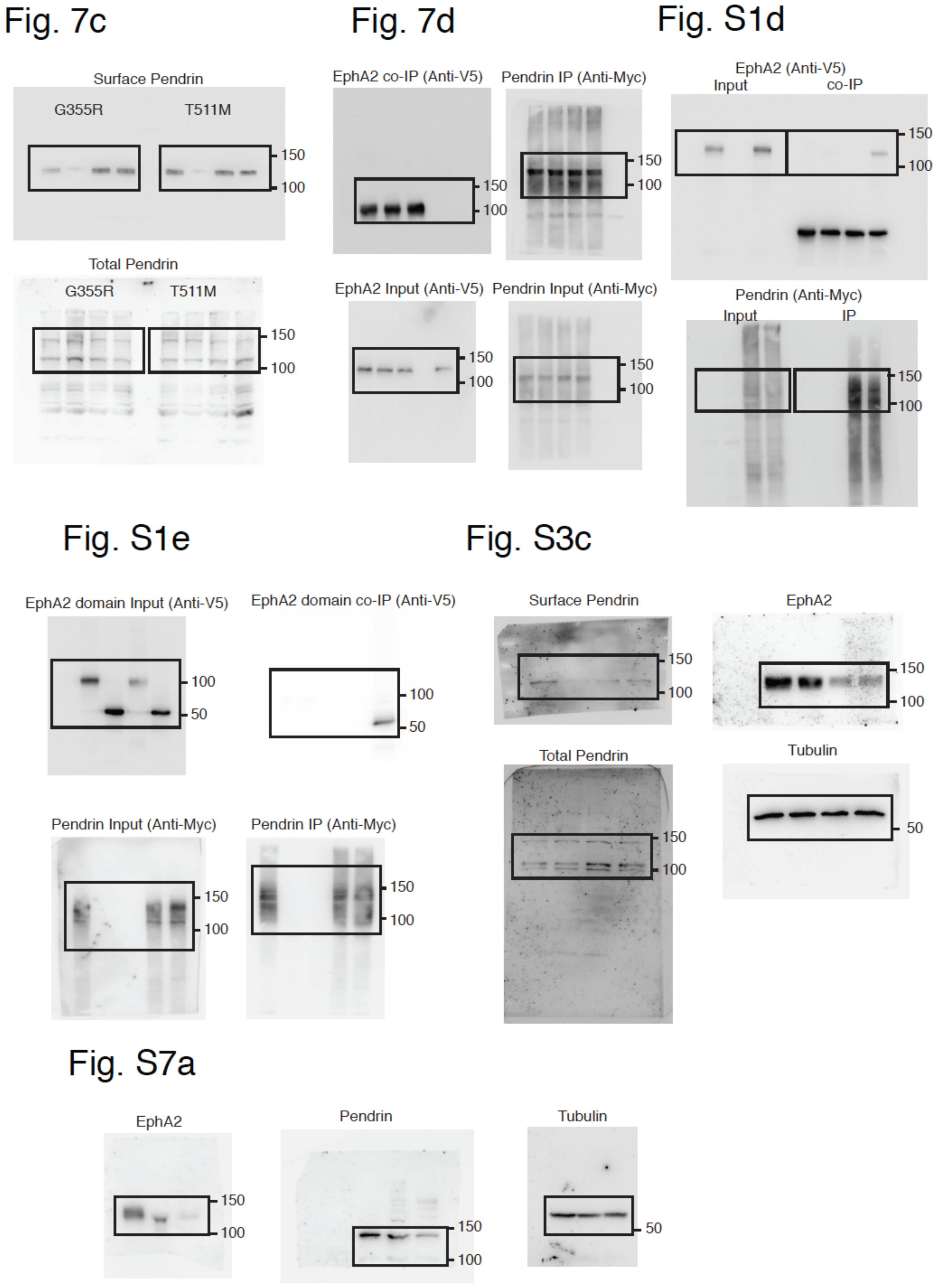

Supplementary Figure 9. Full blot of western blotting analysis in the figures
