# Supplementary material for: Digenic inheritance of mutations in EPHA2 and SLC26A4 in Pendred syndrome
Source: Nat Commun. 2020 Mar 12;11:1343. doi: 10.1038/s41467-020-15198-9 (PMC7067772; doi:10.1038/s41467-020-15198-9)
Supplement: Supplementary file 3 — Reporting Summary [file 41467_2020_15198_MOESM3_ESM.pdf]

nature research

Corresponding author(s): Masanori Nakayama

Last updated by author(s): Jan 13, 2020

# Reporting Summary

Nature Research wishes to improve the reproducibility of the work that we publish. This form provides structure for consistency and transparency in reporting. For further information on Nature Research policies, see Authors & Referees and the Editorial Policy Checklist .

## Statistics

For all statistical analyses, confirm that the following items are present in the figure legend, table legend, main text, or Methods section.

| n/a | Confirmed | |
|---|---|---|
| [ ] | [x] | The exact sample size (*n*) for each experimental group/condition, given as a discrete number and unit of measurement |
| [ ] | [x] | A statement on whether measurements were taken from distinct samples or whether the same sample was measured repeatedly |
| [ ] | [x] | The statistical test(s) used AND whether they are one- or two-sided<br>*Only common tests should be described solely by name; describe more complex techniques in the Methods section.* |
| [x] | [ ] | A description of all covariates tested |
| [x] | [ ] | A description of any assumptions or corrections, such as tests of normality and adjustment for multiple comparisons |
| [ ] | [x] | A full description of the statistical parameters including central tendency (e.g. means) or other basic estimates (e.g. regression coefficient) AND variation (e.g. standard deviation) or associated estimates of uncertainty (e.g. confidence intervals) |
| [x] | [ ] | For null hypothesis testing, the test statistic (e.g. *F*, *t*, *r*) with confidence intervals, effect sizes, degrees of freedom and *P* value noted<br>*Give P values as exact values whenever suitable.* |
| [x] | [ ] | For Bayesian analysis, information on the choice of priors and Markov chain Monte Carlo settings |
| [x] | [ ] | For hierarchical and complex designs, identification of the appropriate level for tests and full reporting of outcomes |
| [x] | [ ] | Estimates of effect sizes (e.g. Cohen's *d*, Pearson's *r*), indicating how they were calculated |

*Our web collection on statistics for biologists contains articles on many of the points above.*

## Software and code

Policy information about availability of computer code

| | |
|---|---|
| Data collection | NDP.view2, Leica Application Suite v2.8.1, Image Reader LAS-4000 |
| Data analysis | Volocity v6.3, Prism 5 v5.0f, NDP.view2, Protter, Primer3Plus, ImageJ v1.51a |

For manuscripts utilizing custom algorithms or software that are central to the research but not yet described in published literature, software must be made available to editors/reviewers. We strongly encourage code deposition in a community repository (e.g. GitHub). See the Nature Research guidelines for submitting code & software for further information.

## Data

Policy information about availability of data

All manuscripts must include a data availability statement. This statement should provide the following information, where applicable:
- Accession codes, unique identifiers, or web links for publicly available datasets
- A list of figures that have associated raw data
- A description of any restrictions on data availability

All data are available from the corresponding author on reasonable request.

# Field-specific reporting

Please select the one below that is the best fit for your research. If you are not sure, read the appropriate sections before making your selection.

[x] Life sciences [ ] Behavioural & social sciences [ ] Ecological, evolutionary & environmental sciences

For a reference copy of the document with all sections, see nature.com/documents/nr-reporting-summary-flat.pdf

# Life sciences study design

All studies must disclose on these points even when the disclosure is negative.

| | |
|---|---|
| Sample size | No statistical methods were used to predetermine sample sizes. Sample sizes were determined based on previous experience to obtain statistical significance and reproducibility. |
| Data exclusions | No data was excluded. |
| Replication | All attempts at replication were successful. |
| Randomization | Randomization was not performed because laboratory animals were allocated based on their genotypes. |
| Blinding | Blinding was not carried out. |

# Reporting for specific materials, systems and methods

We require information from authors about some types of materials, experimental systems and methods used in many studies. Here, indicate whether each material, system or method listed is relevant to your study. If you are not sure if a list item applies to your research, read the appropriate section before selecting a response.

## Materials & experimental systems

| n/a | Involved in the study | |
|---|---|---|
| ☐ | ☒ | Antibodies |
| ☐ | ☒ | Eukaryotic cell lines |
| ☒ | ☐ | Palaeontology |
| ☐ | ☒ | Animals and other organisms |
| ☐ | ☒ | Human research participants |
| ☒ | ☐ | Clinical data |

## Methods

| n/a | Involved in the study | |
|---|---|---|
| ☒ | ☐ | ChIP-seq |
| ☒ | ☐ | Flow cytometry |
| ☒ | ☐ | MRI-based neuroimaging |

## Antibodies

| | |
|---|---|
| Antibodies used | Anti-Mouse EphA2 - R&D Systems Cat # AF639<br>Anti-Human / Mouse EphA2- Invitrogen Cat # 347400<br>Anti-Human / Mouse Pendrin - NOVUS Cat # NBP1-60106<br>Anti-Human / Mouse Pendrin -LSBIO Cat # LS-B6627<br>Anti-Human / Mouse / Rat ephrin-B2 - R&D Systems Cat # AF496<br>Anti-Human / Mouse / Rat ephrin-A1- Invitrogen Cat # 34-3300<br>Anti-Mouse / Rat KCNJ10 - Alomone Labs Cat # APC-035<br>Anti-Human / Mouse / Rat Phospho-EphA2 - Cell Signaling Cat # 12677S<br>Anti-Human / Mouse / Rat V5 Tag - Invitrogen Cat # R960-25<br>Anti-Mouse / Rat myc (clone 9E10) - Santa Cruz Cat # 626803<br>Anti-Human / Mouse / Rat a Tubulin (clone B-5-1-2) - Sigma Cat # T5168<br>Anti-Human / Mouse EEA1 (N-19) - Santa Cruz Cat # SC-6415 |
| Validation | All commercial antibodies have been tested for specificity by their respective suppliers. |

## Eukaryotic cell lines

Policy information about cell lines

| | |
|---|---|
| Cell line source(s) | Madin-Darby Canine Kidney II cells (MDCK II), Human embryonic kidney cells kidney cells 293 T (HEK293T) cells were obtained from ATCC |
| Authentication | Identity of the MDCK II, HEK293 and HEK293T cell lines were frequently checked by their morphological features, but have not been authenticated. |
| Mycoplasma contamination | All cell lines were tested negative for mycoplasma contamination. |
| Commonly misidentified lines (See ICLAC register) | No commonly misidentified cell lines were used. |

## Animals and other organisms

Policy information about studies involving animals; ARRIVE guidelines recommended for reporting animal research

| | |
|---|---|
| Laboratory animals | EphA2 was targeted in ESCs via promoter trap experiments using the reverse orientation splice acceptor (ROSA) beta-Geo retroviral gene trap vector. Insertion site for trap vector is located in the first intron of EphA2. These targeted ESCs were then injected into blastocysts of fertilized C57BL / 6 female mice and backcrossed for 5 generations to generate the EphA2 knock-out (EphA2 KO) line. To maintain the EphA2 knock-out mouse line, C57BL/6 EphA2 KO mice were cross with 129SV mice. |
| Wild animals | The study did not involve wild animal. |
| Field-collected samples | The study did not involve samples collected from the field. |
| Ethics oversight | All laboratory animals were handled according to the regulation of Federation for Laboratory Animal Science Associations (FELASA), and under the oversight of a local officer. |

Note that full information on the approval of the study protocol must also be provided in the manuscript.

## Human research participants

Policy information about studies involving human research participants

| | |
|---|---|
| Population characteristics | The patient carrying c.1300 G >A of SLC26A4 is 22 years old female and presented congenital bilateral sensorineural hearing loss, goitre and skin disorders. Another patient carrying c.1229C>A of SLC26A4 is 14 years old female and exhibited progressive, symmetrical sensorineural hearing loss and goitre. |
| Recruitment | Study participants are all affected by congenital sensorineural hearing loss with Enlarged Vestibular Aqueduct (EVA) and diagnosed by computerized tomography scan and repeated auditory examinations. Each participant voluntarily participates in the study based on fully being informed. |
| Ethics oversight | Study was carried out under the oversight of local ethics committee. |

Note that full information on the approval of the study protocol must also be provided in the manuscript.
